# Performance of PVDF Based Membranes with 2D Materials for Membrane Assisted-Crystallization Process

**DOI:** 10.3390/membranes11050302

**Published:** 2021-04-21

**Authors:** Mirko Frappa, Francesca Macedonio, Annarosa Gugliuzza, Wanqin Jin, Enrico Drioli

**Affiliations:** 1 Institute on Membrane Technology, National Research Council of Italy (CNR-ITM), via Bucci 17/C, 87036 Rende, Italy; m.frappa@itm.cnr.it (M.F.); e.drioli@itm.cnr.it (E.D.); 2 State Key Laboratory of Materials-Oriented Chemical Engineering, College of Chemical Engineering, Nanjing Tech University, 30 Puzhou Road (S), Nanjing 211816, China; wqjin@njtech.edu.cn; 3 Department of Environmental and Chemical Engineering, University of Calabria, via Bucci Cubo 44A, 87036 Rende, Italy

**Keywords:** membrane crystallization, graphene, bismuth telluride

## Abstract

Membrane crystallization (MCr) is a promising and innovative process for the recovery of freshwater from seawater and for the production of salt crystals from the brine streams of desalination plants. In the present work, composite polymeric membranes for membrane crystallization were fabricated using graphene and bismuth telluride inks prepared according to the wet-jet milling (WJM) technology. A comparison between PVDF-based membranes containing a few layers of graphene or bismuth telluride and PVDF-pristine membranes was carried out. Among the 2D composite membranes, PVDF with bismuth telluride at higher concentration (7%) exhibited the highest flux (about 3.9 L∙m^−2^h^−1^, in MCr experiments performed with 5 M NaCl solution as feed, and at a temperature of 34 ± 0.2 °C at the feed side and 11 ± 0.2 °C at the permeate side). The confinement of graphene and bismuth telluride in PVDF membranes produced more uniform NaCl crystals with respect to the pristine PVDF membrane, especially in the case of few-layer graphene. All the membranes showed rejection equal to or higher than 99.9% (up to 99.99% in the case of the membrane with graphene). The high rejection together with the good trans-membrane flux confirmed the interesting performance of the process, without any wetting phenomena, at least during the performed crystallization tests.

## 1. Introduction

Throughout the world, the valorisation and recovery of waste from other separation processes are of increasing importance to reduce environmental pollution and promote circular economy. Membrane crystallization (MCr) offers a robust and simple solution for the sustainable separation and/or purification of valuable components in the pharmaceutical, fine chemicals, food and biochemical industries [1,2,3,4]. The driving force of the crystallization process is the partial vapour pressure difference through the membrane sides. Liquid feed evaporates at the surface of a hydrophobic membrane and passes through the membrane pores. The hydrophobic nature of the used membranes blocks the passage of liquid through them [1,2,5,6]. The continuous distillation process concentrates the feed solution until the supersaturation level is reached and, in turn, crystal formation and growth occur. In order to have fast nucleation and crystals with good properties, the membrane structure plays an important role. Membrane modification or special coating might be done to enhance the mass transfer through the membrane thus improving the MD/MCr performance [7,8,9,10,11]. In this regard, graphene flakes, due to their attractive hydrophobic and anti-wetting nature, anti-fouling properties, and selective sorption of water vapours, were used for the preparation of composite PVDF membranes [12,13,14] and tested in MD and MCr processes [7,12,14,15,16,17,18,19]. Recently, other transition metal dichalcogenide monolayers (TMDCs) are being introduced in PVDF-based membranes to enhance the performance of MD and MCr, too [16,20]. In particular, Bi_2_Se_3_ crystals in PVDF based membranes were studied for the crystallization of NaCl through MCr [20]. The interaction between the graphene or Bi_2_Se_3_ with the interface solution was established providing a reduction in nucleation time and an increase in the growth rate of the crystals.

In contrast with graphene, TMDCs are relatively new materials in the field of desalination. In general, TMDCs are semiconductors of the type MX2, where M is a transition metal atom and X is a chalcogen atom. Usually, TMDCs are composed by layers combined with each other, layer by layer, with van der Waals force, and the weak interaction between layers influences the properties of the bulk TMDCs significantly [21]. Unlike the most commonly studied 2D material (i.e., graphene), TMDCs have an intrinsic direct bandgap, and are promising in applications such as electronic components, in desalination or water treatment devices, and others [7,15,17,22,23].

The objective of this work is to test hydrophobic membranes with Bi_2_Te_3_ flakes (BT) confined in PVDF matrix in MCr process. As far as we know, PVDF membranes with bismuth telluride have never been prepared and tested in the MCr process. Bi_2_Te_3_ is a topological insulator material that has attracted strong recent interest due to its many remarkable properties [24,25,26,27,28]. Topological insulators have currently emerged as one of the most actively researched subjects in condensed matter physics. Moreover, the bulk of a topological insulator possesses an insulating gap, and the thermal conductivity of Bi_2_Se_3_ and Bi_2_Te_3_ are 0.4 and 1.6 W m^−1^ K^−1^, respectively, against a value of about 4000 W m^−1^ K^−1^ for graphene. This is a particularly useful and interesting property for the membrane crystallization process, for which membranes with low thermal conductivity are required in order to reduce heat losses by conduction.

A comparative study is proposed between membranes with graphene flakes (G) and Bi_2_Te_3_ flakes (BT) exfoliated by wet-jet milling (WJM) technique. For the crystallization test, 5 M NaCl solution was used as feed. Whenever possible, the composite membranes prepared in this work have been compared with the PVDF composite membrane with Bi_2_Se_3_ used in [20], although it was not possible to give a complete and direct comparison due to the very different operating conditions used in this work compared to those utilized in [20] (i.e., lower feed temperature, lower temperature gradient, lower feed flow rate).

At the end, we will assess if the integration of a few of layers of material can provide an enhancement of the membrane performance in the crystallization of sodium chloride.

## 2. Experimental Section

### 2.1. Materials

PVDF (Solef^®^6020, Solvay Solexis: water adsorption <0.040% @23 °C after 24 h; dp = 1.78 kg m^−3^) was kindly provided by Solvay Specialty Polymers (Milan, Italy). Three different types of N-Methyl-2-pyrrolidone (NMP) inks with few-layers 2D materials (Bi_2_Te_3_ and graphene) were kindly supplied by Graphene Labs, Fondazione Istituto Italiano di Tecnologia (Genova, Italy) (Table 1). Then, 2-propanol (IPA, WWR PROLAB: d = 0.78 kg∙m^−3^) was used as non-solvent for membranes preparation. Fluorinert (FC-40, Novec (from Sigma-Aldrich, Bollate, Italy) was used for gas-liquid displacement measurements for pore size and overall porosity estimation. NaCl with a degree of purity of 100% was purchased from VWR International S.r.l. (Milan, Italy).

### 2.2. Membrane Preparation

The membranes were prepared by dry-wet phase inversion technique according to the same procedure detailed in previous works [15,18]. In particular, 12 wt.% of PVDF powder was added under mechanical stirring to the dispersions containing the 2D materials. The final concentration of the nanofillers in the membranes was either 0.5% or 7% calculated with respect to the polymer for each 2D materials (Table 1). Each mixture was uniformly cast on a glass plate by using a casting knife regulated on 250 μm (Elcometer Instruments Inc. Manchester, England). The casting solution was successively coagulated in a bath containing IPA in order to promote the precipitation of the polymer and the formation of flat porous membranes. The latter were washed in milli-Q water, air-dried at room temperature overnight and annealed at 30 °C for 1 h before using.

### 2.3. Membrane Characterization

The composite PVDF membranes were characterized in order to analyse their morphology, and to measure their porosity, pore size distribution, mechanical proprieties, hydrophobic character and thickness.

Membrane morphology was inspected using scanning electron microscope (SEM, Zeiss-EVO MA10, Oberkochen, Germany). Small samples specimens were broken in liquid nitrogen and placed in a sample holder. An ultra-thin coating of electrically-conducting gold (Au) was deposited by using a sputter coating for SEM and the morphology of the membranes was analysed in high vacuum.

The hydrophobicity of the composite membranes was characterized by measuring water contact angle using CAM 200-KSV instrument LTD (Helsinki, Finland). Ultra-pure water droplets (filtered by USF ELGA plant, High Wycombe, UK) with a volume of 0.4 μL were dropped onto the membrane surface at room temperature. The images were captured by a digital camera allowing apparent static contact angles to be measured. Each sample was measured at five different positions and the average value was calculated.

Pore size and pore size distribution were determined through porometer (Capillary Flow Porometer-CFP 1500 AXEL, Porous Materials Inc., Ithaca, NY, USA). The mean pore size was estimated according to the gas-liquid displacement technique. Three samples with an effective area of 3.5 cm^2^ were filled by FC-40 and the liquid was displaced from bigger to smaller pores with increasing pressure. The overall porosity was measured by filling them with FC-40. The membrane weight was estimated before and after filling, and the porosity was expressed in percentage as the ratio between the volume occupied by the fluorinert liquid and the volume of the membrane. The procedure was repeated on six specimens.

Mechanical properties (i.e., tensile stress and elongation at break) were investigated through the tensile elongation testing. The membranes were cut to a predetermined length of 5 cm and clamped to a tensile stress-strain meter Roell/Zwick universal testing machine, single-column model Z2.5 (Genova, Italy). Each sample was stretched with a constant rate of 5 mm/min, as described in [29]. Elongation at break (*ε*) and tensile stress (*σ*) were determined by the following equations:

(1) $$\varepsilon =\frac{L-L_{0}}{L_{0}}\cdot 100$$

(2) $$\sigma =\frac{F}{A_{c}}$$

where *L* is the length of the PVDF-based membranes until the break, *L*_0_ the length of the membranes sample in natural extension state, *F* the force, and *A_c_* the area of cross-section.

Transmission electron microscopy (TEM) analysis was performed by drop-casting the 2D material dispersion onto ultrathin C-film on holey carbon 400 mesh Cu grids, from Ted Pella Inc (Redding, CA, USA). The graphene and bismuth telluride samples were diluted 1:50. The grids were stored under vacuum at room temperature to remove the solvent residues. TEM images were captured by a JEOL JEM-1011 transmission electron microscope (Peabody, MA, USA), operated at an acceleration voltage of 100 kV.

X-ray photoelectron spectroscopy analysis was accomplished using a Kratos Axis UltraDLD spectrometer (Manchester, UK) on samples drop-cast onto gold-coated silicon wafers. The X-ray photoelectron spectroscopy (XPS) spectra were acquired using a monochromatic Al Kα source operating at 20 mA and 15 kV. The analyses were carried out on a 300 × 700 μm^2^ area. High-resolution spectra of C 1s and Au 4f peaks were collected at pass energy of 10 eV and energy step of 0.1 eV. Energy calibration was performed setting the Au 4f_7/2_ peak at 84.0 eV. Data analysis was carried out with CasaXPS software version 2.3.17 (Casa Software Ltd., Teignmouth, UK).

### 2.4. Membrane Crystallization Experiments

Membrane crystallization experiments were executed in Direct Contact (DC) configuration using high concentrated NaCl solution (5 M) as feed and distillate water as permeate.

Feed and permeate were recirculated in the plant with a flow rate of 250 and 100 mL∙min^−1^ respectively; and with a temperature of 34 ± 0.2 °C at the feed side and 11 ± 0.2 °C at the permeate side, respectively. Retentate and distillate streams were converged, in a counter-current way, toward the membrane module where the liquid water was evaporated. On the retentate side, a pump was taking and sending the heated feed to the membrane module. Moreover, on the distillate side, a second pump ensured the counter-current recycle of the cold stream in order to remove from the solution the vapour diffusing through the membrane pores. The trans-membrane fluxes were estimated by evaluating the weight variations in the distillate tank. The salt conductivity of the feed and permeate were measured by using a conductivity meter (HI 2300 bench meter supplied by Hanna Instruments, Woonsocket, Rhode Island, USA).

Trans-membrane flux was calculated as:

(3) $$J=\frac{Q}{A\cdot t}$$

where J is the permeate flux (L∙m^−2^h^−1^), Q is the permeate volume (*L*) collected during time t (h) and A is the effective area of the membrane (m^2^).

Samples of the feed solution of almost 5 mL were accurately extracted from the retentate side and observed using an optical microscope (Nikon Eclipse LV100ND, Nikon Eclipse LV100ND, Firenze, Italy) in order to determine crystal size distribution and growth rate at different stages of experimentation for all the analysed conditions. In particular, samples containing NaCl crystals were removed from the retentate solution after regular intervals of 30 min from onset of crystallization and each experiment was continued for 60 min to follow the growth of the crystals. Therefore, each crystallization experiment required a time equal to the sum (1) of the time necessary to reach supersaturation and the first clearly visible crystals, plus (2) the 60 min required to observe the growth of the crystals. The evolution of the particle size distribution as a function of time allowed the evaluation of the quality (in terms of coefficient of variation (*CV*) and length to width ratio). Coefficient of variation (*CV*) is a parameter indicating the dispersion of a distribution around the average crystal size. *CV* was calculated using the following equation:

(4) $$CV=\frac{PD_{84\%}-PD_{16\%}}{2\cdot PD_{50\%}}\cdot 100$$

where *CV* is expressed as percentage and PD is the crystal length at the indicated percentage.

Growth rate (*G*) was estimated on the basis of the Randolph-Larson model [4,20] as follows:

(5) $$\ln\left(n\right)=\frac{-L}{Gt}+ln\left(n^{0}\right)$$

where *n* is the crystal population density, *L* is the crystal size, *t* is retention time and *n*^0^ is population density at *L* equal to zero. A plot of ln(*n*) versus *L* is a straight line whose intercept is ln(*n*^0^) and whose slope is −1/Gt. Thus, from a given product sample of known slurry density and retention time, it is possible to obtain the nucleation rate and growth rate for the conditions tested when the sample satisfies the assumptions of the derivation and yields a straight line.

The evolution of particle size distribution as a function of time allows for the evaluation of the nucleation rate (*B*^0^) according to the following equation:

(6) $$B^{0}=n^{0}G$$

## 3. Results and Discussion

### 3.1. Membrane Preparation and Characterizations

The PVDF-based membranes prepared in this work, together with the used nanofillers and related concentration, are reported in Table 1. As it can be observed, composite membranes with BT concentration of 0.5% and 7% were prepared and tested in MCr, and only one with G concentration equal to 0.5% (where 0.5% and 7% represent the final concentration of the filler in the dried membranes with respect to the polymer).

The choice of preparing membranes with filler concentrations equal to 0.5% and 7% derives from the results obtained in some previous works [7,15,19]. Five different PVDF composite membranes with graphene platelets (GP) as filler were prepared in [19]. In particular, the PVDF/GP composite membranes had an increasing GP concentration: 0.33%, 0.5%, 5.0%, 10%, and 20%. The membranes were tested in MD (that is, the antechamber process of membrane crystallization). The obtained results showed that the PVDF/GP composite with 0.5% of filler was the best performing membrane while the PVDF/GP composite with 10% was the worst one.

In [7], PVDF/GP composite membranes with GP concentration of 0.5%, 5.0% and 10% were prepared and tested in MCr: PVDF/GP 5% was the membrane with the highest flux, followed by PVDF/GP10% and PVDF/GP0.5%. However, PVDF/GP5% was the membranes with the highest nucleation and crystal growth rate, followed by PVDF/GP 0.5% and PVDF/GP 10%. PVDF/GP 0.5% was also the membrane with the lowest time for detection of the first small crystals and with the largest number of cubic NaCl crystals, followed by PVDF/GP 5% and PVDF/GP 10%. The research findings suggested to continue the experimentation with a GP concentration equal to 0.5 and in between 5% and 10%.

Moreover, in [15], PVDF composite membranes with graphene produced via WJM (and referred as FLG-WJM) were prepared and compared with PVDF composite membranes with graphene produced via exfoliation based on ultrasonic waves (UW) (and referred as GPNs-UW). It was proved that the inclusion of FLG (0.5%) exfoliated via WJM (FLG-WJM) into the PVDF matrix improved the resistance at break by up to 175% compared to the pristine membrane (PVDF), whereas the resistance at the break increased by 166% for the composite membranes prepared with GPNs-UW at the same concentration (0.5%) with respect to the pristine PVDF membrane. Moreover, when the graphene percentage in the WJM membranes was increased up to 7.0% an improvement of 38% was obtained while a drastic reduction in the elongation at break (−73%) was estimated for GPNs-UW at 10% nanofiller. Therefore, the largest size of FLG improved the elastic behaviour of the composite membranes with respect both to the pristine PVDF membrane and to the composite membranes prepared with GPNs-UW. The membranes were tested in MD and, in term of flux, FLG-WJM (0.5%) was again the best performing membrane among the tested ones.

In the present work, the previous best performing membrane (i.e., FLG-WJM (0.5%) = PVDF/G (0.5%)) was tested for the first time in MCr and was compared (1) with composite PVDF membranes with Bi2Te3 flakes and (2) with pristine PVDF membrane. As concentration of the filler, the results achieved in [7,15,19] suggested to use 0.5% and 7% for Bi_2_Te_3_ and 0.5% for graphene.

**Figure 1:**
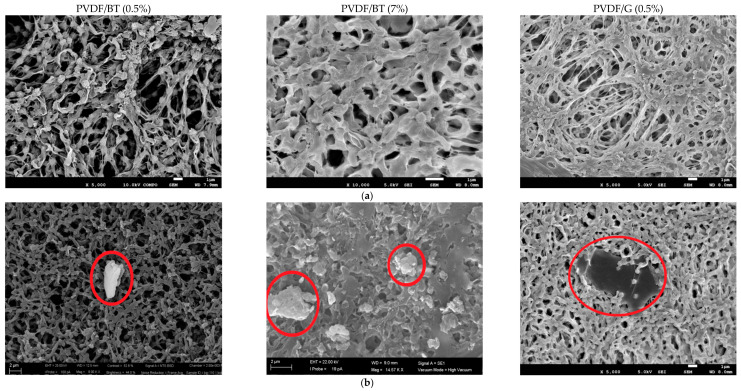
(**a**) SEM micrographs collected on the top surface of PVDF/BT (0.5%), PVDF/BT (7%) and PVDF/G (0.5%) membranes; (**b**) 2D fillers enclosed in the various membrane networks.

The SEM micrographs showed the spherulitic-like morphology of the polymer networks (in agreement with delayed solvent–nonsolvent demixing mechanisms which direct the formation of a particulate-like porous architecture) and the 2D materials randomly entrapped in the matrix (Figure 1).

**Figure 2:**
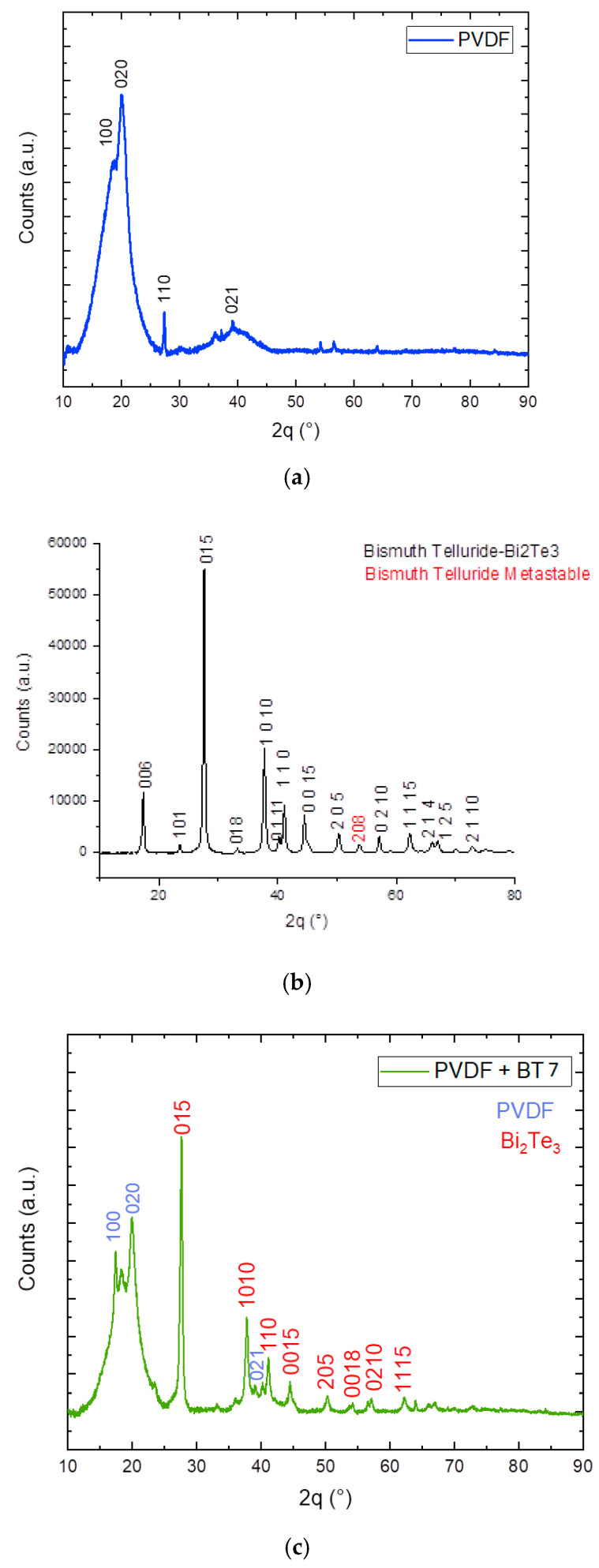
XRD patterns for (**a**) pristine PVDF, (**b**) Bi_2_Ti_3_ flakes and (**c**) PVDF/BT (7%) membrane.

The presence of 2D flakes in membrane matrix was also confirmed by X-ray diffraction analysis (XRD) where the typical peaks corresponding to the used 2D fillers were observed (Figure 2c). In particular, Figure 2 shows the comparison between XRD patterns of the pristine PVDF membrane (without filler) and of the Bi_2_Ti_3_ flakes, with the PVDF membrane with high concentration of bismuth telluride (7%). When the concentration of 2D materials inside the membrane is low (0.5%), XRD analysis cannot relieve the presence of 2D materials in PVDF matrix.

**Figure 3:**
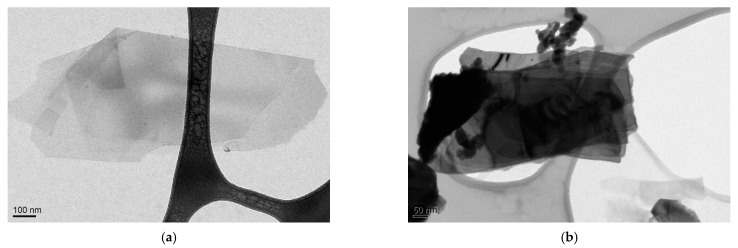
TEM micrographs for (**a**) graphene flakes and (**b**) bismuth telluride flakes exfoliated via WJM.

TEM images for selected graphene and bismuth telluride flakes exfoliated via WJM are shown in Figure 3a,b, respectively. They confirmed the presence of few layers of Bi_2_Te_3_ (BT) and graphene (G) in the solvent residues. Graphene flakes are larger and more regular than Bi_2_Te_3_ flakes (which, on the contrary, show irregular and wrinkled geometries). In particular, graphene and Bi_2_Te_3_ flakes have a lateral size of about 490 and 200 nm, respectively. Moreover, the darkest contrast of Bi_2_Te_3_ flakes with respect to graphene flakes is characteristics of a thicker structure.

Wet-jet milling (WJM) is, in fact, a technique allowing to produce few-layer flakes with larger lateral size than compared to that from other techniques, e.g., exfoliation by ultrasonic waves (UW) [19,30]. For example, in the case of graphene, usually, the lateral size of the exfoliated flakes is lower (around 120 nm), while the thickness is similar to that obtained by WJM [15,31,32]

All the prepared membranes showed good hydrophobic proprieties with contact angle values equal or higher than 128° (Table 2). The thickest membrane was the one with high concentration of Bi_2_Te_3_ (7%). On the contrary, at low filler concentration, membranes of comparable thickness were obtained. A bit scattering can be appreciated for the porosity of the membranes. PVDF pristine membrane exhibited the highest porosity. The presence of 2D materials reduced the porosity: PVDF/BT (0.5%) and PVDF/BT (7%) showed porosity of 75% and 77%, respectively, higher than the membrane with graphene that was the least porous membrane among those prepared. Mean pore size showed an analogous trend: the smallest mean pore size was detected in the PVDF/G (0.5%) membrane (0.29 μm) while the largest in the PVDF pristine membranes (0.52 μm). The graphene flakes tend to penetrate in membrane pore reducing the pore size and the porosity of the membrane respect to PVDF membrane [10]. The inclusion of graphene flakes during the membrane preparation produces much more viscous casting solutions, which hinders the solution de-mixing process during the phase inversion resulting in lower pore size and membrane porosity [32]. In the case of the composite membranes PVDF/BT (0.5%) and PVDF/BT (7%), the presence of Bi_2_Te_3_ reduces pore size and porosity compared to the pristine PVDF membrane, however the effect is much less pronounced than the membrane with graphene. In fact, in the case of Bi_2_Te_3_, membranes morphology still displays a high degree of accessible free gaps even when a large amount of Bi_2_Te_3_ is embedded in the polymer matrix (PVDF/BT (7%) with porosity around 77 ± 1%). This agrees with what has already been observed in [20] in the case of composite membrane with another TMDC, that is the case of a PDVF membrane with Bi_2_Se_3_, where the presence of the filler reduced porosity by only 6% (from 73.4% to 69%).

**Figure 4:**
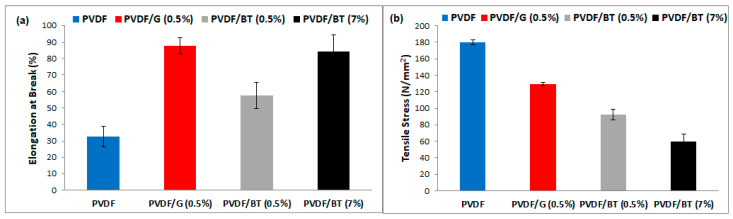
Elongation at break (**a**) and tensile stress (**b**) estimated for pristine PVDF, PVDF/G (0.5%), PVDF/BT (0.5%) and PVDF/BT (7%) membranes.

Histograms reported in Figure 4a showed that the inclusion of 2D materials exfoliated via WJM into the PVDF matrix improves the resistance at break in comparison to the PVDF pristine membrane. This property is a fundamental requisite for membrane durability and processing. The highest resistance at break was measured with PVDF/G (0.5%) and PVDF/BT (7%) membranes. The enhanced mechanical resistance of the WJM membranes was further confirmed by the trend of Young modulus, which appeared to be more contained when the 2D fillers are embedded in the polymer matrix, especially for the membrane PVDF/BT (7%) (Figure 4b). The 2D nature of the nanoflakes is the major reason for enhancement of mechanical proprieties due to major specific surface area of 2D flakes and improved mechanical interlocking/adhesion at the filler-matrix interface [16,33]. In the literature, contradictory results can be found (worsening or enhancing of elastic moduli) depending on the effect of the average lateral size, directional alignment, degree of dispersion in polymer matrix, and the thickness of the 2D nanofillers on the final properties of polymeric matrices [34,35].

### 3.2. Membrane Crystallization Tests

**Figure 5:**
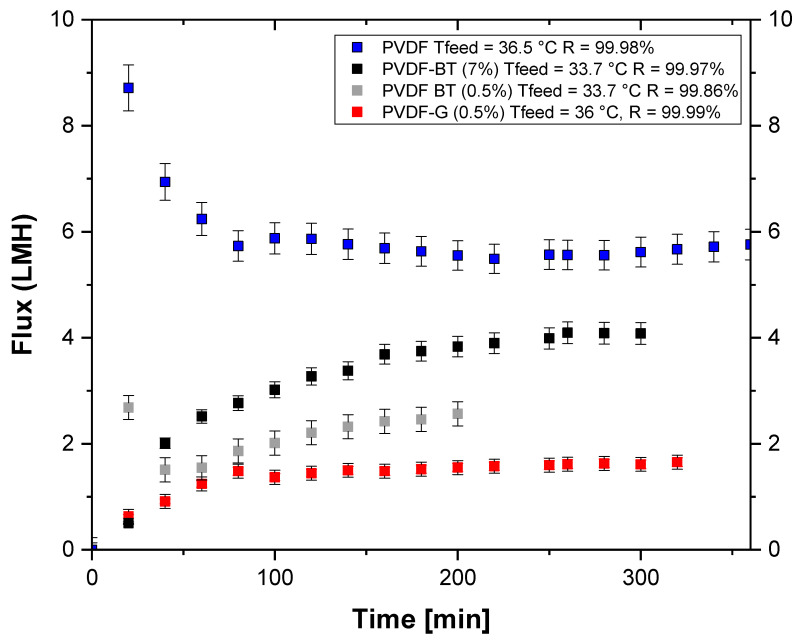
Trend of the flux as a function of time for PVDF-based membranes functionalized with 2D materials at (0.5%) and PVDF/BT (7%).

Figure 5 shows the trend of trans-membrane flux with time for the three membranes functionalized with 2D materials and for the pristine PVDF membrane. The membranes were tested utilizing almost the same operating conditions. PVDF pristine membrane exhibited the highest trans-membrane flux (average flux of about 5.7 L∙m^−2^h^−1^) due to its higher porosity and mean pore size with respect to the other membranes. Among the PVDF membranes functionalized with 2D materials, PVDF/BT (7%) presented the highest trans-membrane flux (average flux of about 3.9 L∙m^−2^h^−1^). This result was due to its higher porosity (77%) and mean pore size (0.5 μm) with respect to the others. Moreover, PVDF/BT (7%) is also the thickest membrane, resulting in an increase of the resistance to mass transport on the one hand, while on the other decreases the heat loss due to conduction [36]. For similar considerations, the other two membranes PVDF/BT (0.5%) and PVDF/G0.5% exhibited an average flux of about 2.7 L∙m^−2^h^−1^ and 1.6 L∙m^−2^h^−1^, respectively. All the membranes showed rejection equal (PVDF/BT (0.5%)) or higher than 99.9% (PVDF/G (0.5%)) where rejection equal to 99.99% was measured. The high values of rejection obtained in the tests guaranteed that, at least in the experimental time, the salts infiltration through the membrane pores was negligible and that the analyzed membranes preserved the crucial requisite of hydrophobicity.

Moreover, the continuous removal of pure water as permeate from the feed solution induced the saturation of the feed and the formation of sodium chloride crystals.

The obtained NaCl crystals showed in most cases cubic form according to the expected geometry of crystallized NaCl. The *CV*, *G*, and *B_0_* values related to the performed tests are reported in Table 3. Our results indicate a more uniform distribution of NaCl crystals with the PVDF/G (0.5%) membrane, which exhibits *CV* values ranging from 36.7% to 44.2% (Table 3) within the 60 min of crystals growth observation (i.e., from sample 1 to sample 3). PVDF/BT (0.5%) showed also good *CV* values, with decreasing CV from sample 1 (time with clearly visible crystals in the feed solution) to sample 3 (i.e., 60 min later). This trend was due to the fact that, in the chosen observation time, the most part of crystals are growing (as proved by the decreasing values of B_0_ and increasing values of middle diameter d_m_) from sample 1 to sample 3. The worst CV values were obtained with the PVDF/BT (7%) membrane due to the simultaneous presence of low values of G and high values of B_0_. This indicates that the formation of new crystals takes place in the solution rather than the growth of those previously formed. This creates a wide crystal size distribution (and high *CV* = 65.4%).

For what concerns NaCl size, crystals of comparable dimension were obtained (after one hour of growth) with the three membranes with 2D materials (16.27 μm, 16.82 μm and 16.70 μm for PVDF/G (0.5%), PVDF/BT (0.5%) and PVDF/BT (7%), respectively). Larger NaCl crystals were measured in the case of pristine PVDF membrane due to its highest observed crystal growth G (7.95 × 10^−5^ mm min^−1^). However, a wider distribution (confirmed by the high CV values) was also measured with such PVDF membrane.

**Figure 6:**
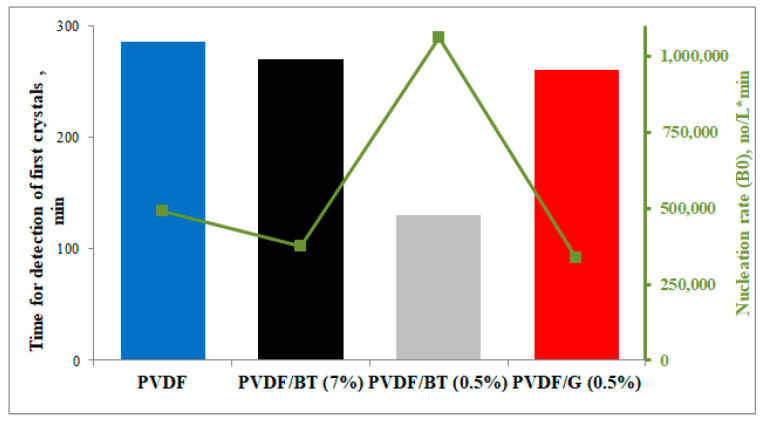
Time for detection of the first crystals and nucleation rate for the PVDF-pristine membrane and PVDF functionalized membranes.

Moreover, the presence of fillers in PVDF-based membranes reduced the time for detection of the first clearly visible crystals in comparison with PVDF-pristine membrane, from 285 min to 140 min in the case of PVDF/BT (0.5%) (Figure 6).

The obtained experimental results agree with the findings of Perrotta et al. [7,17], where it was suggested that well-established interactions at the graphene–solution interface stimulate water sequestration from ion–water clusters and promote ion–ion aggregation. As a consequence, reduced nucleation time and increased growth rate of the crystals can be detected in the PVDF/G (0.5%) with respect to the pristine PVDF membrane (sample 1, Table 3). Moreover, in [20], it was proven that PVDF composite membranes with Bi_2_Se_3_ promote the capture of water molecules by adsorption thus favoring a faster achievement of the saturation conditions in the membrane with the TMDC with respect to the pristine PVDF membrane. It was proposed that the rapid water removal produces a shrinking of the ionic core preceding the crystallization, in analogy to theoretical predictions for NaCl crystallization from aqueous solutions [37]. An analogous behavior is proposed to occur, in this work, in the case of composite membranes with Bi_2_Te_3_.

The sudden formation of crystals with the PVDF/BT (0.5%) membrane (Figure 6), and the choice to continue each crystallization test for 60 min after the formation of the first clearly visible crystals, explain the reduced experimental time of the test with the PVDF/BT (0.5%) membrane compared to the others (Figure 5).

**Figure 7:**
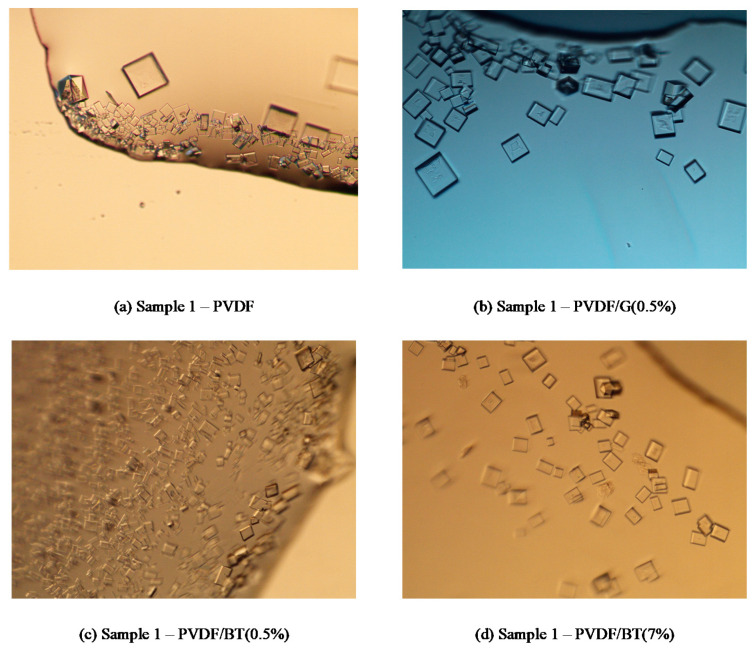
Pictures collected on the first sample of NaCl crystals obtained with the different analysed membranes: (**a**) PVDF, (**b**) PVDF/G (0.5%), (**c**) PVDF/BT (0.5%) and (**d**) PVDF/BT (7%).

Figure 7 shows the comparison, in sample 1, between NaCl crystals obtained with the four different analysed membranes. In agreement with the results reported in Table 3, it is possible to observe the numerous but small crystals obtained with PVDF/BT(0.5%) (where *B_0_* = 1060979 and *d_m_* = 9.56 μm), the slightly less numerous but large and different crystals obtained with PVDF (where *B_0_* = 490593, *d_m_* = 20.6 μm and *CV* = 77.1%), the even less numerous and small (*B_0_* = 337756 and *d_m_* = 13.38 μm) but more uniform (*CV* = 47%) crystals obtained with PVDF/BT (7%), and the relatively few but larger and more uniform crystals obtained with PVDF/G(0.5%) (where *B_0_* = 280475, *d_m_* = 18.92 μm and *CV* = 36.7%).

**Figure 8:**
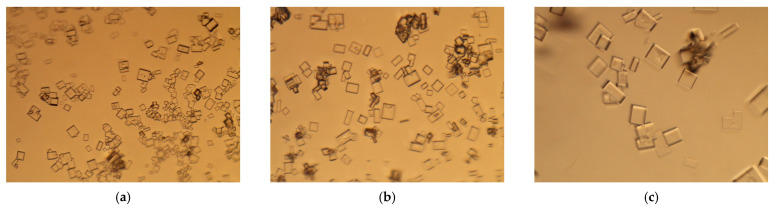
Pictures collected on different samples of NaCl crystals obtained with PVDF/BT (0.5%) membrane (magnification 20×): after (**a**) 140, (**b**) 170 and (**c**) 200 min.

Figure 8 clearly shows the growth in size, from the first to the last sample, of the NaCl crystals as achieved with the PVDF/BT (0.5%) membrane (again in agreement with the trend reported in Table 3.

**Figure 9:**
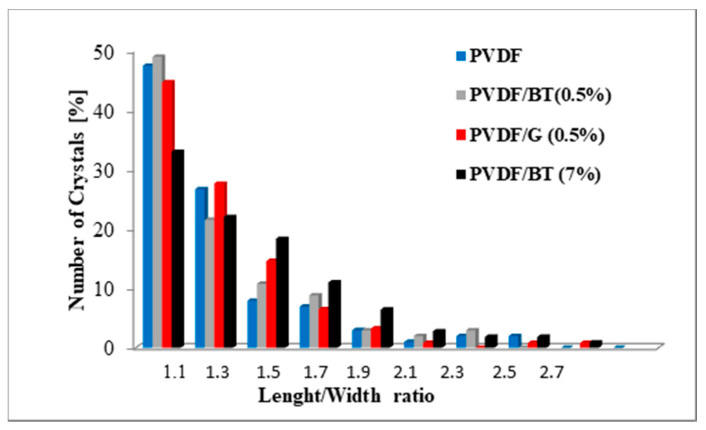
Percentage of crystals in function of length/width ratio for the pristine and functionalized PVDF-based membranes.

Figure 9 yields a clear indication regarding disparate crystals size distribution and shape uniformity based on the number of crystals as a function of the ratio between length and width for the different prepared membranes. The most part of crystals showed the characteristic cubic block-like form in accordance with the expected geometry of the NaCl crystals, particularly in the case of PVDF/BT (0.5%).

## 4. Conclusions

Three different composite hydrophobic PVDF-based membranes with 2D nanofillers were prepared via the phase-inversion method. Dispersions of graphene and bismuth telluride at two different concentrations (0.6 and 10 g∙L^−1^) were chosen as 2D nanofillers sources for a final concentration in the membrane of 0.5% for the graphene and 0.5% and 7% for bismuth telluride. The suitability of the prepared membranes for membrane assisted crystallization process was analysed by crystallizing sodium chloride starting from 5M NaCl aqueous solutions. Under the same operating conditions, PVDF/BT (7%) membrane exhibited flux higher than the other 2D composite membranes, (3.9 L∙m^−2^h^−1^) while PVDF/BT (0.5%) and PVDF/G (0.5%) showed an average flux of 2.7 L∙m^−2^h^−1^ and 1.6 L∙m^−2^h^−1^, respectively. The confinement of graphene and bismuth telluride in polymeric hydrophobic matrices has produced a more uniform NaCl crystals dispersion (especially in the case of PVDF/G (0.5%) membrane) and reduced the time for detection of the first clearly visible crystals (from 285 min in the case of PVDF-pristine membrane to 140 min in the case of PVDF/BT (0.5%)). Moreover, the high rejection together with a good trans-membrane flux confirmed the interesting performance of the process, without any wetting phenomena, at least during the crystallization tests.

The experimental evidence suggests a new possible use of TMDC within polymeric structures. The results obtained indicate that these new materials can actually be used in innovative membrane processes related to water desalination and mineral recovery.

## Figures and Tables

**Table 1:** List of prepared membrane with details about utilized filler.

Name of Membrane	Filler Dispersed in NMP
PVDF/BT (7%)	Bi_2_Te_3_ 10 g∙L^−1^
PVDF/BT (0.5%)	Bi_2_Te_3_ 0.6 g∙L^−1^
PVDF/G (0.5%)	Graphene 0.6 g∙L^−1^

**Table 2:** Morphological parameters estimated for tailored pristine and PVDF/2D materials membranes.

Membrane	Contact Angle (°)	Thickness (μm)	Mean Pore Size (μm)	Porosity (%)
PVDF	139 ± 3	71 ± 2	0.52 ± 0.05	82 ± 4
PVDF/G (0.5%)	136 ± 1	62 ± 3	0.24 ± 0.05	56 ± 7
PVDF/BT (0.5%)	128 ± 8	68 ± 1	0.50 ± 0.2	75 ± 1
PVDF/BT (7%)	130 ± 2	100 ± 5	0.50 ± 0.08	77 ± 1

**Table 3:** NaCl crystal parameters obtained with PVDF-based membranes.

	**PVDF**				**PVDF/G (0.5%)**			
	**CV**	**B0**	**G (mm∙min^−1^)**	**d_m_ (μm)**	**CV**	**B0**	**G (mm∙min^−1^)**	**d_m_ (μm)**
Sample 1	77.1	490,593	0.0000298	20.6	36.7	280,475	0.0000385	18.92
Sample 2	48.4	257,598	0.0000524	42.5	43.8	199,025	0.0000456	23.25
Sample 3	53.8	149,088	0.0000795	65.1	44.2	374,721	0.0000251	17.27
	**PVDF/BT (0.5%)**				**PVDF/BT (7%)**			
Sample 1	54.2	1,060,979	0.0000317	9.56	46.0	337,756	0.0000239	13.38
Sample 2	44.4	1,633,024	0.0000315	12.08	29.5	343,006	0.0000245	13.93
Sample 3	43.1	811,706	0.0000388	16.82	65.4	392,031	0.0000212	16.70
